# Hepatic Arterial Infusion Chemotherapy Followed by Lipiodol Infusion for Advanced Hepatocellular Carcinoma with Portal Vein Tumor Thrombus: A Single-Center Experience

**DOI:** 10.3390/medicina57080779

**Published:** 2021-07-30

**Authors:** Kuan-Ting Chen, Kun-Feng Tsai, Henry W. C. Leung, Agnes L. F. Chan, Shyh-Yau Wang, Huei-Lung Liang, Sheng-Yeh Tang, Chu-Kuang Chou, Hsin-Yu Chen, Shan-Ho Chan, Ming-Feng Li

**Affiliations:** 1 Department of Radiology, Taichung Armed-Forces General Hospital, Taichung 411, Taiwan; cocacolas310131@gmail.com; 2 Department of Radiology, Kaohsiung Veterans General Hospital, Kaohsiung 813, Taiwan; hlliang@vghks.gov.tw; 3 School of Medicine, National Defense Medical Center, Taipei 114, Taiwan; 4 Gastroenterology and Hepatology Section, Department of Internal Medicine, An Nan Hospital, China Medical University, Tainan 709, Taiwan; tsai.kf@gmail.com (K.-F.T.); Jethroleaf@gmail.com (S.-Y.T.); 5 Department of Medical Sciences Industry, Chang Jung Christian University, Tainan 711, Taiwan; 6 Department of Radiation Oncology, An Nan Hospital, China Medical University, Tainan 709, Taiwan; hwclmd@yahoo.com; 7 Department of Pharmacy, An Nan Hospital, China Medical University, Tainan 709, Taiwan; agnes.lf@gmail.com; 8 Department of Radiology, An Nan Hospital, China Medical University, Tainan 709, Taiwan; D23930@mail.tmanh.org.tw; 9 Department of Medical Imaging and Radiology, Shu-Zen Junior College of Medicine and Management, Kaohsiung 821, Taiwan; shchan@ms.szmc.edu.tw; 10 Division of Gastroenterology and Hepatology and Clinical Trial Center, Ditmanson Medical Foundation Chia-Yi Christian Hospital, Chia-Yi 600, Taiwan; vacinu@gmail.com; 11 Division of Nephrology, Department of Internal Medicine, Kaohsiung Veterans General Hospital, Kaohsiung 813, Taiwan; xychen16@gmail.com

**Keywords:** hepatocellular carcinoma, hepatic arterial infusion chemotherapy, lipiodol infusion, portal vein tumor thrombosis

## Abstract

*Background and Objectives*: To evaluate the effectiveness of hepatic arterial infusion chemotherapy (HAIC) followed by lipiodol infusion in advanced hepatocellular carcinoma (HCC) with portal vein tumor thrombus (PVTT). *Materials and Methods*: Thirty-two patients with advanced HCC and PVTT who received HAIC with regimens of cisplatin, mitomycin-C, and 5-fluorouracil followed by lipiodol infusion were enrolled. The primary efficacy endpoint was tumor response rate. The modified Response Evaluation Criteria in Solid Tumors (mRECIST) was used for assessment of treatment response. The secondary endpoints were overall survival (OS) and progression free survival (PFS). Prognostic factors for survival also were evaluated. *Results*: The median OS and PFS were 11.9 and 9.5 months, respectively. Seventeen patients (53.1%) achieved objective response, and 23 patients (71.9%) achieved disease control. The length of survival in the responder and disease control groups was longer than in the non-responder and progressive disease groups after two cycles of HAIC (responder vs. non-responder: 16.5 vs. 7.9 months, *p* = 0.001; disease control vs. progressive disease: 12.3 vs. 5.6 months, *p* < 0.001) and after completing HAIC (responder vs. non-responder: 15.7 vs. 6.9 months, *p* = 0.001; disease control vs. progressive disease: 13.6 vs. 6.9 months, *p* < 0.001). Better survival was associated with Child-Pugh A liver function (*p* = 0.013), with early response to two HAIC cycles (*p* = 0.009), and with response (*p* = 0.02) and disease control (*p* = 0.001) after completing HAIC treatment. *Conclusion:* HAIC followed by lipiodol infusion is a safe and feasible treatment for advanced HCC with PVTT. Patients with early response could continue HAIC treatment with expected prolonged survival.

## 1. Introduction

Hepatocellular carcinoma (HCC) is the second most common cause of cancer death in the world [1,2]. In Taiwan, more than 90% of HCC is related to chronic viral infection, most often to hepatitis B virus (HBV) carriers comprising of 2.5 million people, and hepatitis C (HCV) carriers comprising of 0.7 million people [3]. In the early stage of HCC, various treatment modalities, including surgical resection, radiofrequency ablation (RFA), and liver transplantation, can achieve five-year-survival rates of 50–70% [4,5]. However, only 30% of HCC patients benefit from curative treatment because HCC is most often diagnosed in the intermediate and advanced stages of disease [6].

Portal vein tumor thrombosis (PVTT) is present in about 10% to 40% of HCC patients at the time of diagnosis [7]. PVTT increases portal venous pressure, which may lead to variceal bleeding, ascites, hepatic encephalopathy, liver failure, or other fatal sequelae. PVTT involving the main portal trunk or the first-order branches usually is a contraindication to transarterial embolization (TAE) because inadequate perfusion of the liver results in acute hepatic failure with hepatic encephalopathy and potentially death [7]. Patients who have advanced HCC with PVTT survive only 2–4 months with supportive treatment [7,8]. In unresectable HCC, sorafenib treatment was found to improve the median overall survival in the Sorafenib Hepatocellular Carcinoma Assessment Randomized Protocol (SHARP) trial [9] and the Asia–Pacific trial [10].

In the treatment of HCC with hepatic arterial infusion chemotherapy (HAIC), response rates of 12.2–52% have been reported [11], and HAIC has been reported to have better tumor response rates and survival benefit than sorafenib alone [12]. A randomized clinical trial demonstrated that patients with advanced HCC treated with the combination of sorafenib and HAIC had significantly better overall survival, higher response rate, and longer median progression-free survival than did patients treated with sorafenib alone [13].

Most HAIC studies have used only anticancer agents, usually 5- fluorouracil (5-FU) and cisplatin. Studies from our institute used 5-FU, cisplatin, mitomycin C, and leucovorin, with resultant response rates of 18–20% [14,15]. Moreover, HAIC non-responders have short survival time, giving limited opportunity to try other treatments when HAIC has failed. In our pilot study, we found that HAIC followed by lipiodol infusion appeared to be more efficacious than HAIC monotherapy for HCC, but data on the combination of HAIC treatment and lipiodol infusion in HCC with PVTT are scarce [16,17]. This study aimed to evaluate the effectiveness of HAIC followed by lipiodol infusion in the treatment of advanced HCC with PVTT.

## 2. Materials and Methods

### 2.1. Patient Population

This prospective observational cohort study was approved by the Institutional Research Board of An Nan Hospital, China Medical University, and written informed consent was obtained from each patient. From May 2016 to December 2019, 32 consecutive patients with advanced HCC and PVTT were enrolled. The inclusion criteria were: (a) tumor size ≥ 8 cm in diameter; (b) not suitable for operation; (c) platelet counts > 50,000 cells/mm^3^; (d) prothrombin time, international normalized ratio (INR) < 1.5; (e) peripheral white cell counts > 2500 cells/mm^3^; and (f) Child-Pugh class liver function A or B. All patients received at least two cycles of HAIC therapy. Patients with extrahepatic metastases were also included because extrahepatic metastases were not uncommon among those who had large tumor burden and PVTT. Exclusion criteria were patients with Child-Pugh C liver function or serum total bilirubin > 5 mg/dL, i.e., severe liver dysfunction.

### 2.2. Temporary Infusion System

The temporary infusion system used was previously described [14]. Briefly, after skin preparation and local anesthetic injection, the left subclavian artery was punctured using the Seldinger technique. A temporary catheter (4 or 5 Fr) was placed for diagnostic angiography to enable individually customized treatment and delivery of HAIC. The gastroduodenal and right gastric arteries were embolized with microcoils through a microcatheter to prevent reflux of chemo-agents into the stomach and duodenum, and a follow-up hepatic arteriography was performed to verify the embolization. The tip of the catheter was placed at the common or proper hepatic artery. For patients with relatively small or torturous hepatic arteries, micro catheters were used to deliver anticancer agents. Patients were asked to cough and take a deep breath to determine if the catheter tip would migrate. After placement of the temporary catheter, low-dose heparin was continually infused to prevent catheter-related thrombus formation.

### 2.3. Regimen of Chemotherapy and Lipiodol Infusion

The administration of HAIC in this study was continuous method, and agents suitable for continuous HAIC include anthracycline-based agents; mitomycin C; fluorouracil (5-FU), which are time dependent. Cisplatin also has a synergistic effect as a modulator of 5-FU [18]. Therefore, the regimen chosen in this study was cisplatin, mitomycin-C and 5-FU, which was in accordant with our previous studies [14,15]. The chemotherapeutic course was 20–30 min infusion of cisplatin (10 mg/m^2^) and mitomycin-C (2 mg/m^2^) in 50 mL isotonic sodium chloride solution a day for five days. In addition, 100 mg/m^2^ of 5-FU in 250 mL of isotonic sodium chloride solution was administered for 24 h via an infusion pump for five days. Leucovorin (15 mg/m^2^) was given daily to enhance the effect of 5-FU.

After the fifth day of chemotherapy infusion, all patients were brought back to our angio-suite for lipiodol infusions. The rationale to infuse lipiodol as a single agent following chemotherapy infusion was to facilitate and enhance anticancer agent retention in the target tissues while achieving tumor embolization at the same time. Lipiodol was given via the temporary catheter until stasis of the tumor-feeding artery was reached without obvious reflux. For tumors less than 10 cm in size, 6 to 10 mL of lipiodol was infused, and for tumors 10 to 20 cm in size, 10–20 mL of lipiodol was infused. Lipiodol infusion was always performed under continuous fluoroscopy to prevent ectopic embolism from happening. Finally, the catheter was removed followed by direct compression to achieve hemostasis. The treatment intervals were adjusted according to the patients’ liver function or other signs of toxicity after each HAIC, ranging from four to six weeks. The treatment was terminated when patients’ clinical conditions were not suitable for further HAIC.

After the HAIC treatment, additional therapies were applied if necessary, which depends on the treatment responses, performance status, and hepatic function. Additional treatment included targeted therapy with sorafenib, immunotherapy, external radiation therapy or surgical treatment.

### 2.4. Study Assessment

The primary efficacy endpoints were objective response rate and disease control. Response was evaluated after two cycles of HAIC and after completing HAIC. The secondary endpoints were overall survival (OS) and progression-free survival (PFS). OS was defined as time from first treatment to the last visit or the death of the patient; PFS was the defined as time from first treatment to disease progression according to image study. The modified Response Evaluation Criteria in Solid Tumors (mRECIST) was used for assessment of treatment response after at least two cycles of HAIC. The objective response rate was defined as complete response (CR) + partial response (PR); disease control rate was defined as objective response rate + stable disease (SD). Pre-treatment surveys included medical history, physical examination, complete blood count, blood chemistry, virologic marker, serum alpha-fetoprotein (AFP), computed tomography (CT), magnetic resonance imaging (MRI), or positron emission tomography scan before. During treatment, the patients’ adverse responses were evaluated with Common Terminology Criteria for Adverse Events (CTCAE) v. 4.0. All patients who completed the treatment course were followed with liver function test, AFP, sonography and/or three-phase CT or MRI liver scan after every two-treatment course.

### 2.5. Statistics

OS and PFS were estimated with the Kaplan-Meier method. Log-rank statistics were used to compare survival curves. To identify the prognostic factors associated with patient survival, Cox’s regression model univariate analyses and multivariate analysis with were conducted with proportional hazards; results were presented as hazard ratio (HR) and 95% confidence intervals (CIs). A *p*-value < 0.05 was set as the threshold for significance. Statistical analysis was performed with SPSS 22 (Released 2013. IBM SPSS Statistics for Windows, Version 22.0. IBM Corp. Armonk, NY, USA).

## 3. Results

### 3.1. Patient Characteristics

Patients’ clinical characteristics are listed in Table 1. The median age was 64 years (range, 40–84), and 26 patients (81.3%) were male. The most common cause of underlying chronic hepatitis was viral hepatitis (78.1%), most often chronic hepatitis B (59.4%). Although all patients had PVTT and Barcelona Clinic Liver Cancer Stage C (100%), most patients had a Child-Pugh class A liver function (84.4%). Five (15.6%) patients had extrahepatic metastasis, all of which were pulmonary metastases. Nineteen (59.4%) patients began sorafenib treatment before HAIC treatment. Three patients underwent TACE, and two underwent TACE and RFA before HAIC treatment.

### 3.2. Treatment Efficacy

**Figure 1:**
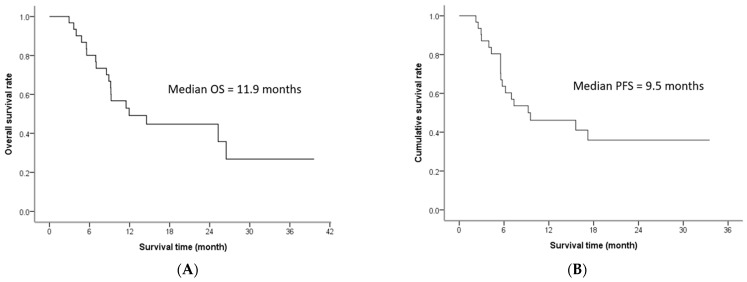
Overall survival (**A**) and progression-free survival (**B**). OS: overall survival; PFS: progression-free survival.

The patients received a total of 108 cycles of HAIC, with all Child-Pugh class A liver function patients receiving a median of 3 HAIC cycles (range, 2–8 cycles) and all five Child-Pugh class B liver function patients receiving only 2 HAIC cycles due to unrecovered hepatic function after HAIC. The median OS in all patients was 11.9 months (range, 1.7–39.6 months), and the PFS was 9.5 months (range, 1.7–33.5 months), as shown in Figure 1. The OS rates in all patients at six months, one year, and two years were 80%, 49% and 45%, respectively. Post-treatment response after two cycles of HAIC, response after completing HAIC, and overall treatment response were given in Table 2.

**Figure 2:**
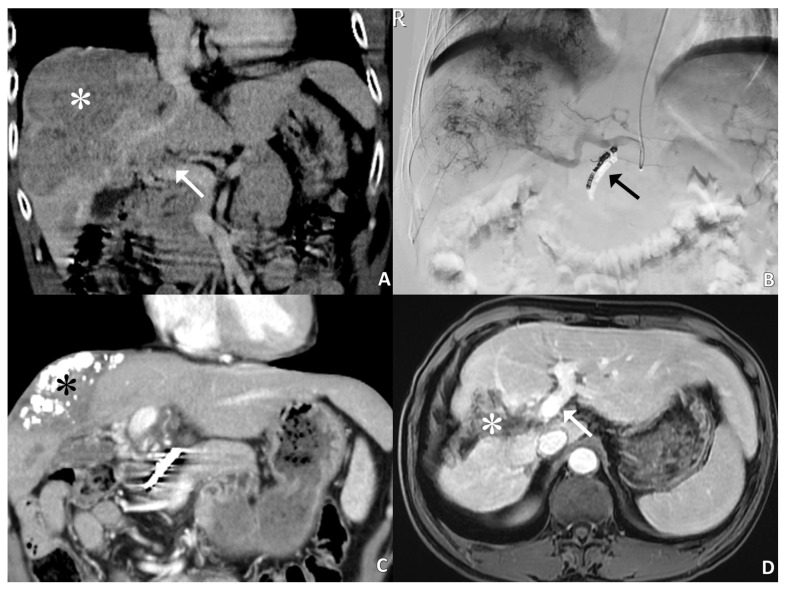
A 55-year-old male patient with complete response after five cycles of HAIC. (**A**): Coronal CT image shows a large HCC (white asterisk) in the right lobe of the liver with main portal venous tumor thrombosis (arrow). The initial serum AFP level is 35,678 ng/mL. (**B**): Hepatic angiogram reveals large tumor burden. The gastroduodenal artery is embolized with microcoils (arrow). (**C**): Partial response is obtained after two cycles of HAIC treatment. The hyperattenuated region (black asterisk) within the tumor is lipiodol retention. (**D**): Follow-up MRI after 5 cycles of HAIC treatment shows tumor shrinkage with cystic necrosis and lipiodol retention (white asterisk) in addition to complete resolution of portal venous tumor thrombosis (arrow). The serum AFP level is 2.5 ng/mL, and the patient has survived for more than 3 years after initial diagnosis of advanced HCC with PVTT.

A representative case of huge HCC with main portal vein thrombosis achieved complete response after treatment, as shown in Figure 2. After two cycles of HAIC, 16 patients (50%) had an objective response, and 25 patients (78.1%) had disease control. Based on the response after completing HAIC, the objective response rate was 53.1% and disease control rate was 71.9%.

**Figure 3:**
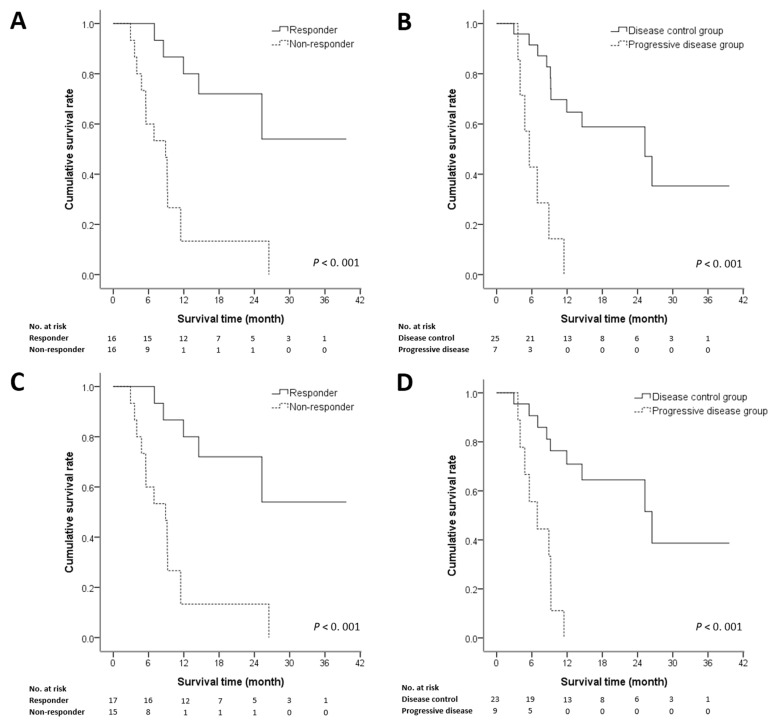
Overall survival of the objective response and disease control groups. (**A**,**B**): After two cycles of hepatic arterial infusion chemotherapy (HAIC) followed by lipiodol infusion; (**C**,**D**): After completing HAIC.

After two cycles of HAIC, the responder group had a higher median OS (16.5 months) than that of the non-responder group (7.9 months) (log-rank test: χ^2^ = 12.01, *p* = 0.001) (Figure 3A). The disease control group had a higher median OS (12.3 months) than did the progressive disease group (5.6 months) (log-rank test: χ^2^ = 13.78, *p* < 0.001) (Figure 3B). The OS rates of early responders to two HAIC cycles at six months, one year, and two years were 100%, 80%, and 72%, respectively, which is better than the rates of non-responders (60%, 13% and 13%, respectively). These trends were present also after completing HAIC: Responders had a higher median OS (15.7 months) than did non-responders (6.9 months) (log-rank test: χ^2^ = 11.36, *p* = 0.001) (Figure 3C), and the disease control group had a higher median OS (13.6 months) than did the progressive disease group (6.9 months) (log-rank test: χ^2^ = 13.52, *p* < 0.001) (Figure 3D). The OS rates of responder after completing HAIC at six months, one and two years were 100%, 81% and 73%, respectively, which was better than the rates of non-responders (57%, 11% and 11%, respectively).

### 3.3. Prognostic Factors of Survival

**Figure 4:**
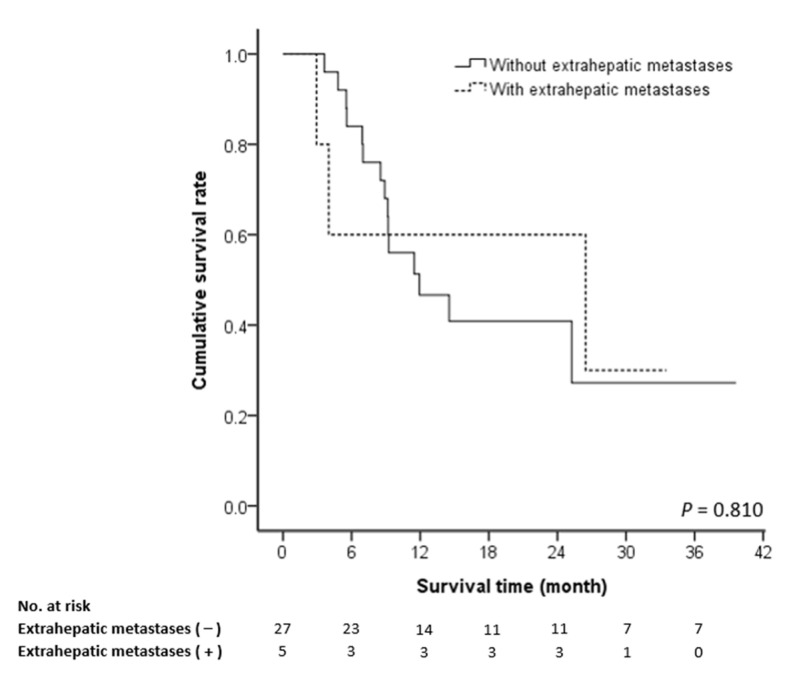
Cumulative survival rate according to the presence of extrahepatic metastasis.

The prognostic factors affecting patient survival were analyzed according to pre-treatment and post-treatment variables, as illustrated in Table 3. The univariate and multivariate analyses revealed only Child-Pugh score (A vs. B) to be a pre-treatment prognostic factor (HR = 0.28, 95% CI: 0.1–0.76; *p* = 0.013). Extrahepatic metastasis was not a prognostic factor to survival (HR = 0.86, 95% CI: 0.24–3.08; *p* = 0.81) (Figure 4), and pre-treatment with sorafenib was not associated with survival (HR = 1.10, 95% CI: 0.34–3.55; *p* = 0.87).

The post-treatment variables were analyzed after two cycles of HAIC and after completing HAIC (Table 3). Multivariate analysis revealed that the objective tumor response after two cycles (HR = 0.1, 95% CI: 0.03–0.35; *p* = 0.009) and the objective response (HR = 0.13, 95% CI: 0.02–0.74; *p* = 0.02) and disease control after completing HAIC (HR = 0.17, 95% CI: 0.02–0.31; *p* = 0.001) were independent predictors for longer survival.

### 3.4. Complications and Adverse Effects

The adverse effects that occurred during treatment are summarized in Table 4. There was no treatment-related mortality. The most common adverse effects were grade 1/2 nausea or vomiting, fever, and elevated liver enzymes. The side effects were tolerable, nonfatal, and treatable with conservative medication. Among patients who experienced grade 3/4 adverse events, symptom-relieving medication controlled most. Among patients who had elevated liver enzymes and hyperbilirubinemia during HAIC treatment, infusion of chemotherapy was stopped. One patient had a subclavian artery pseudoaneurysm, which was treated with endovascular stent grafting. No other vascular complications, including occlusion or vasculitis of the hepatic artery, were recognized.

### 3.5. Cause of Death

Twenty-two (68.8%) of the 32 patients died during the follow-up. The most common cause of death was progression of intra-hepatic tumor (72.7%). One patient (4.5%) died from hepatic dysfunction, two (9.1%) died from variceal bleeding, two (9.1%) died from infection, and one (4.5%) died from an unknown cause. Ten patients (31.3%) were alive at the end of follow-up.

## 4. Discussion

The prognosis of advanced HCC patients with PVTT is poor, with reported survival to be 2–4 months under conservative treatment [7]. Treatment of advanced HCC with PVTT is a major challenge, and there have been no satisfactory treatment until now. The present study strengthened the combination of HAIC and lipiodol infusion in the treatment of advanced HCC patients with PVTT—response rate 53.1%, median OS 11.9 months, and disease control rate 71.9%. The 1- and 2-year OS rates were 49% and 45%, respectively. The survival in responder and disease control group was significantly better than non-responder and progressive disease group after two cycles of HAIC and after completing HAIC. As for prognostic factors of survival, patients with Child-Pugh A liver function had better survival than did those with B liver function, whereas other pre-treatment factors did not affect patient survival. Positive post-treatment prognostic factors were treatment responders after two cycles of HAIC, and treatments responders and disease control group after completing HAIC treatment. Since data regarding the combination of HAIC and lipiodol infusion are scarce [16,17], we believe that our experience in treating advanced HCC with PVTT with this combination of agents is clinically valuable.

Most previous HAIC studies only applied anticancer agents, showing unsatisfactory response rate, as with previous studies from our institute which revealed low response rate with 18–20%, median survival of 8–9.5 months, and survival rates at one and two years of 29% and 14% for advanced HCC patients with PVTT [14,15]. This study, based on the similar criteria of patient enrollment, technique and chemotherapy regimen as our prior studies, shows superior response rate of 53.1%, median survival time of 11.9 months, and survival rates at one and two years of 49% and 45% after addition of lipiodol infusion, confirming efficacy of combination of HAIC and lipiodol infusion. In the limited experience of others, a group from Japan reported a better response rate of 75% and median survival time of 32 months in advanced HCC patients treated with lipiodol combined with anticancer agents compared with a response rate of 35% and median survival time of 10.2 months with anticancer agents alone [16,19]. The combination of anticancer agent and lipiodol is thought to be more effective through two possible mechanisms: tumor artery embolization and prolonged retention of the anticancer agent in the target tissues by lipiodol [20]. Therefore, applying HAIC followed by lipiodol infusion could enhance treatment efficacy.

Considering pre-treatment prognostic factors, patients with Child-Pugh A liver reserve had better OS than did patients with Child-Pugh B; other factors, including patient age, initial tumor size, main portal vein invasion, extra-hepatic metastasis, and initial serum AFP level, were not associated with survival. Patients with Child-Pugh A liver function were able to undergo more than two courses of treatment, whereas those with Child-Pugh B liver function had deteriorated and unrecovered hepatic function after receiving two courses of treatment, prompting cessation of chemotherapy infusion. This observation is consistent with those of studies purportedly showing that HCC patients with Child–Pugh class A liver function benefited from HAIC [15,21,22]. Understandably, patients with better liver reserves can better tolerate adverse events and toxicity of anticancer agents and receive more courses of HAIC [23].

PVTT is found in about 10% to 40% of all HCC patients at the time of diagnosis [7], and has been reported an independent prognostic factor for poor survival under conservative treatment [24,25,26]. However, our study did not find main portal vein invasion an independent prognostic factor, and, in relevant reports, a randomized prospective comparative study found that main portal vein invasion is not a predictive factor affecting OS in advanced HCC patients undergoing HAIC [27]. Invasion of the portal vein trunk did not have prognostic significance [28], nor was the grade of portal vein invasion a factor influencing survival [23,29]. Combination of HAIC and lipiodol infusion in advanced HCC with PVTT is thought to enhance treatment efficacy, and hence PVTT is uncorrelated to patient survival. Furthermore, most of responders of this study had partial or even complete resolution of PVTT after HAIC treatment.

Although HAIC treatment is mainly used for local control of HCC with poor response to extrahepatic metastasis, our study includes patients with extrahepatic metastasis, whereas most previous studies exclude those patients [14,15,16]. Our study showed that existing extrahepatic metastasis was not associated with patient survival, and most patients (72.7%) died of intra-hepatic tumor progression, in line with previous studies [28,30]. Thus, when considering patient eligibility in treatment protocols for advanced HCC, liver reserve appears to be a relevant factor, whereas extrahepatic metastasis, patient age, initial tumor size, serum AFP level, and degree of portal vascular invasion are not.

Our study demonstrated high early response rate (50%) and survival benefit for early responders to two cycles of HAIC. The overall survival rates of early responder at one and two years are 80% and 72%, respectively. If early response can be achieved, patients may be able to continue loco-regional therapies with further benefit from improved OS. Song et al. [28] have reported that responder and disease control group after the second cycle of HAIC were positive prognostic factors for survival, and Lin et al. [31] reported that early response to four-week HAIC in advanced HCC with PVTT was associated with better median OS. Because the life expectancy of advanced HCC patients with PVTT is limited, identifying which patients are early responders is important. Our study suggests that advanced HCC patients who have early response can continue loco-regional therapies and expect favorable outcome, whereas patients without early response should consider combinations of other treatments or shift to other treatment modalities. Furthermore, our study also recognizes that the therapeutic response of HAIC with lipiodol infusion is an important prognostic factor, as a previous report has stated [16].

Our study found that the addition of sorafenib did not improve the survival outcome for advanced HCC patients under HAIC treatment. Based on the SHARP trial, sorafenib has been the standard treatment for advanced HCC patients with PVTT or extrahepatic metastasis [9]. However, this approach has been challenged. According to the meta-analysis by Zhuang et al. in 2019 [12], HCC patients receiving HAIC had better response rate, disease control rate, progression-free survival, and overall survival than did those receiving sorafenib; and a randomized clinical trial found that patients receiving the combination of sorafenib and HAIC had significantly better clinical outcomes than those receiving sorafenib alone [13]. In our study, concurrent sorafenib treatment does not serve as a prognostic factor because the effectiveness of HAIC treatment surpassed the therapeutic effect of sorafenib. Nevertheless, given that sorafenib is the current first-line therapy for advanced HCC with macroscopic vascular invasion or extrahepatic metastases and HAIC is only a locoregional therapy, sorafenib is still recommended with HAIC treatment concurrently in patients with extrahepatic metastases. Moreover, HAIC treatment is also a reasonable option for patients who become refractory or intolerant to sorafenib.

In recent years, molecular targeted therapy, such as lenvatinib, atezolizumab and bevacizumab, has emerged as a new cancer treatment method [32]. However, these treatment agents, except for sorafenib, were not reimbursed by Taiwan National Health Insurance (NHI) during the study period (2016–2019). Hence, these patients had to pay 4000 to 5000 US dollars per month if they wish to receive other new molecular targeted agents. Most of the patients could not afford these new therapeutic drugs, which is why we chose HAIC and sorafenib as the mainstay treatment for advanced HCC with PVTT or extrahepatic metastasis in our study, and the patients receiving new generation immunotherapy or target therapy were excluded.

The treatment of advanced HCC patients with PVTT is still under debate without effective treatment modalities. Current treatments of choice are HAIC, TACE, radioembolization, radiotherapy, target therapy or immunotherapy. Treatment modalities for advanced HCC with macrovascular invasion or PVTT are summarized in Table 5. Shui et al. reported a median OS of 3  ±  1.0 months for advanced HCC patients with PVTT receiving stereotactic body radiotherapy alone [33]. In comparison with sorafenib, lenvatinib, nivolumab or pembrolizumab, radiotherapy and TACE in treating advanced HCC with macrovascular invasion, the potential role of HAIC followed by lipiodol infusion in advanced disease is suggested due to superior treatment efficacy over most of treatments. Salem et al. reported that PVTT patients with Child-Pugh A liver reserve receiving radioembolization had better median OS (Child-Pugh A vs. B: 10.4 months vs. 5.6 months) and response rate (Child-Pugh A vs. B: 50% vs. 28%) than those of Child-Pugh B liver function [34]. The treatment efficacy in Child A group is comparable to our results. Given that this study is single-arm design, randomized controlled clinical trials is needed to verify whether the advanced HCC patients with macrovascular invasion is more suitable for HAIC followed by lipiodol infusion than other treatment modalities.

There are a few limitations to this study. First, the relatively small cohort size and single-arm design without control group were underpowered, which might cause inherent bias and statistical errors. Further randomized controlled studies with more patients are recommended. Second, patients with extrahepatic metastases were included in this study even though HAIC is considered a locoregional therapy effective for intrahepatic tumors. Nevertheless, our study showed extrahepatic metastasis was not related to survival, and the results were comparable to previous studies in which patients with extrahepatic metastases were excluded.

## 5. Conclusions

HAIC followed by lipiodol infusion for treatment of advanced HCC with PVTT is safe and well tolerated. However, its efficacy should be established through randomized, prospective trials before it can become first-line treatment. Early response to two cycles of HAIC is associated with better OS, and advanced HCC patients who have early response can continue HAIC treatment with expected favorable outcome.

## Figures and Tables

**Table 1:** Baseline clinical characteristics.

Patient Characteristics	Statistic
Age (year)	64 (40–84)
Gender (M/F)	26/6
Etiology	
HBV/HCV/HBV + HCV/non-viral	17/6/2/7
Child-Pugh classification (A/B)	27/5
BCLC staging C	32 (100%)
Portal venous thrombosis ^a^	
Vp2/Vp3/Vp4	5/21/6
Maximal tumor size (cm)	
<10/≥10	6/26
Extrahepatic metastasis	5 (15.6%)
PT (INR)	1.04 (0.93–1.26)
Total bilirubin (mg/dL)	0.96 (0.4–3.6)
Albumin (g/dL)	3.8 (2.8–4.7)
Platelet count (×10^3^/mL)	161 (54–469)
Alpha-fetoprotein (ng/mL)	
(≤1000/1000~10,000/>10,000)	18/5/9
Sorafenib	19 (59.4%)
Previous treatment	
(TACE/TACE + RFA/conservative treatment)	3/2/27

Continuous data are presented as the median (range). ^a^ The extent of PVTT was documented according to the Liver Cancer Study Group of Japan classification: Vp0 = no PVTT, Vp1 = segmental portal vein invasion, Vp2 = right anterior/posterior portal vein, Vp3 = right/left portal vein and Vp4 = main trunk. Abbreviations: HBV, hepatitis B virus; HCV, hepatitis C virus; BCLC, Barcelona Clinic Liver Cancer; PT, Prothrombin time; INR, international normalized ratio; RFA, radiofrequency ablation; TACE, transarterial chemoembolization.

**Table 2:** Tumor responses to HAIC followed by lipiodol infusion.

	Response after Two HAIC Cycles, *n* (%)	Response after Completing HAIC, *n* (%)	Overall Treatment Response, *n* (%) ^a^
Complete response	2 (6.3%)	8 (25%)	5 (15.6%)
Partial response	14 (43.8%)	9 (28.1%)	7 (21.9%)
Stable disease	9 (28.1%)	6 (18.8%)	4 (12.5%)
Progressive disease	7 (21.9%)	9 (28.1%)	16 (50%)
Objective response	16 (50%)	17 (53.1%)	12 (37.5%)
Disease control rate	25 (78.1%)	23 (71.9%)	16 (50%)

^a^ Overall treatment response refers to response to all treatment modalities after ceasing HAIC until the end of follow-up. Abbreviation: HAIC, Hepatic arterial infusion chemotherapy.

**Table 3:** Prognostic factors for survival in HAIC followed by lipiodol infusion.

	Crude HR		Adjusted HR ^a^	
	(95% CI)	*p*-Value	(95% CI)	*p*-Value
**Pre HAIC prognostic factor**				
Age (≤65/>65)	0.63 (0.25–1.63)	0.344	0.52(0.13–2.15)	0.369
Sex (F/M)	0.41 (0.15–1.14)	0.087	0.12 (0.01–1.28)	0.079
Tumor size (≤10/>10)	1.1 (0.36–3.38)	0.869	0.75 (0.21–2.62)	0.649
Child-Pugh score (A/B)	0.36 (0.18–0.69)	0.002 *	0.28 (0.1–0.76)	0.013 *
PVTT (nonVp4/Vp4)	1.32 (0.43–4.04)	0.624	0.67 (0.13–3.37)	0.628
Extrahepatic metastasis	0.86 (0.24–3.08)	0.81	0.74 (0.18–3.39)	0.781
AFP level (AFP ≤ 1000/>1000)	0.79 (0.29–2.15)	0.649	0.31 (0.06–1.47)	0.14
Sorafenib	0.68 (0.27–1.71)	0.414	1.1 (0.34–3.55)	0.87
**Post HAIC prognostic factor**				
After two cycles of HAIC				
Objective response				
Responder	0.15 (0.05–0.46)	0.001 *	0.10 (0.03–0.35)	0.009 *
Non-responder	1			
Tumor control				
Control group	0.12 (0.43–0.39)	0.001 *	0.41 (0.04–3.92)	0.44
Progressive group	1			
After completing HAIC				
Objective response				
Responder	0.14 (0.05–0.40)	0.001 *	0.13 (0.02–0.74)	0.02 *
Non-responder	1			
Tumor control				
Control group	0.13 (0.04–0.40)	0.001 *	0.17 (0.02–0.31)	0.001 *
Progressive group	1			

^a^ Adjusted HR: adjusted forage, tumor size, Child-Pugh score, PVTT, extrahepatic metastasis, AFP level and sorafenib in Cox proportional hazards regression. Abbreviations: AFP, Alpha-fetoprotein; HAIC, Hepatic arterial infusion chemotherapy; PVTT, Portal vein tumor thrombosis; HR, Hazard ratio; CI, confidence interval; * *p* < 0.05.

**Table 4:** Adverse events related to treatment.

	Grade 1/2, *n* (%)	Grade 3/4, *n* (%)
Leukopenia	5 (15.6%)	0
Anemia	1 (3.1%)	0
Thrombocytopenia	5 (15.6%)	0
Elevated liver function	8 (25.0%)	1 (3.1%)
Hyperbilirubinemia	5 (15.6%)	2 (6.3%)
Elevated serum creatinine	1 (3.1%)	0
Nausea/vomiting	13 (40.6%)	2 (6.3%)
Diarrhea	2 (6.3%)	3 (9.4%)
Fever	9 (28.1%)	0

**Table 5:** Treatment modalities for advanced HCC with macrovascular invasion or portal vein tumor thrombosis.

Study Reference	Treatment Modality	Patient Number	Response Rate	Disease Control Rate	Overall Survival	Progression Free Survival
This study	HAIC followed by lipiodol infusion	32	53.1%	71.9%	11.9 m	9.5 m
He et al. [13]	Sorafenib	122	5.7%	50.8%	7.13 m	2.6 m
Bruix et al. [35]	sorafenib	108	NR	38.9%	8.1 m	4.1 m
Chuma et al. [36]	Lenvatinib	61	29.3%	61%	6.7 m	3.4 m
Tsai et al. [37]	Nivolumab or pembrolizumab	45	20.6%	41.2%	8.9 m	NR
Zhang et al. [38]	TACE	131	0–32%	0–68%	4.1–6 m	2.4–3.0 m
Salem et al. [34]	Radioembolization with yttrium-90	Child-Pugh A: 35	50%	NR	10.4 m	5.6 m
		Child-Pugh B: 57	28%	NR	5.6 m	5.9 m
Liu et al. [39]	HAIC	181	26–43.8%	46.3–93.8%	5.3–14.9 m	3.3–4.4 m

Abbreviations: HCC: hepatocellular carcinoma; HAIC, Hepatic arterial infusion chemotherapy; TACE: transarterial chemoembolization; m: months; NR: not reported.

## Data Availability

The data presented in this study are available on request from the corresponding author.
